# Oncological Outcomes of Immature Ovarian Teratoma: A 15-Year Report From a Cancer Center

**DOI:** 10.7759/cureus.95934

**Published:** 2025-11-02

**Authors:** Maria Habib, Kheyal Khalil, Zain Tayyab, Hafsa Atiq, Muhammad Usman, Shahid Khattak, Aamir Syed

**Affiliations:** 1 Obstetrics and Gynaecology, Ashford and St Peter's Hospitals NHS Foundation Trust, Surrey, GBR; 2 Surgical Oncology, Shaukat Khanum Memorial Cancer Hospital and Research Centre, Lahore, PAK; 3 Gynae-Oncology, Shaukat Khanum Memorial Cancer Hospital and Research Centre, Lahore, PAK

**Keywords:** adjuvant chemotherapy, cytoreductive surgery (crs), immature ovarian teratomas, oncological outcomes, primary debulking surgery

## Abstract

Background: Immature ovarian teratomas (IOTs) are a rare ovarian tumor, with recurrence rates varying widely depending on age and grade. Thus far, debate exists regarding the management of IOTs, especially of their recurrences.

Objective: To review clinical characteristics and oncological outcomes of immature ovarian teratoma following primary and secondary surgery.

Methodology: This retrospective cohort study was conducted in the department of surgical oncology, Shaukat Khanum Memorial Cancer Hospital & Research Centre, Lahore, Pakistan, between July 2008 and June 2023 (15 years). All patients with pathological confirmation of IOTs were included in the study. Women were excluded if (1) details of baseline characteristics or primary treatment were unavailable, and (2) if there was no follow-up data. Non-probability consecutive sampling technique was used for the recruitment of patients. Patient demographics and disease characteristics were summarized using descriptive statistics. Progression-free survival time and overall survival were calculated from the time of surgery to the date of recurrence or the date of death. The Kaplan-Meier method was used for survival curves. Statistical analysis was performed using Statistical Product and Service Solutions (SPSS, version 23; IBM SPSS Statistics for Windows, Armonk, NY).

Results: Of 37 women, most of the patients belonged to an age group of 21-30 years (N-16, 43.2%). Stage 1a (N-18, 48.6%) and grade III disease (N-12, 32.4%). Laparotomy was performed in 91.9% (N-34) of the patients. The majority of the patients underwent unilateral salpingo-oophorectomy (N-27, 73%). Twenty-seven patients received adjuvant chemotherapy (73%). Relapse occurred in 31.5% (N-13) of the patients. However, the most common site of relapse was the liver (N-5, 13.5%). Secondary cytoreductive surgery was done in eight (61.5%) patients, while the rest of the cohort received adjuvant chemotherapy. Overall survival after a mean follow-up of 43 months was 81%.

Conclusion: IOT occurs most commonly in young women. The most common presentation is early stage but high grade, and fertility-sparing surgery has good oncological outcomes. With secondary cytoreductive surgery and platinum-based chemotherapy, it is possible to salvage most recurrences.

## Introduction

Malignant ovarian germ cell tumors (MOGCTs) comprise <5% of all ovarian tumors. Immature ovarian teratomas (IOTs) are rare malignant germ cell tumors of the ovary, accounting for less than 1% of all tumors of the ovary [1]. They are more prevalent in the adolescent age group, with a peak incidence between the ages of 15-30 years, where fertility preservation is an important issue while managing these tumors. Unlike mature cystic teratomas, IOTs exhibit a different pattern and have immature neuroectodermal tissue [1].Depending on the amount of immature neuroectodermal tissue, they are divided into grades 1-3. The higher the grade, the poorer the prognosis [2].

Majority of the women present in the early stages of the disease; according to the European Society of Medical Oncology (ESMO) guidelines, the management option is fertility-preserving surgery, which includes unilateral salpingo-oophorectomy with complete staging, along with regular follow-ups to monitor recurrence of the disease [3]. Systemic chemotherapy includes platinum-based chemotherapy, most commonly the BEP (bleomycin, etoposide, cisplatin) regimen, for high-grade and advanced-stage cases to reduce the risk of recurrence [4].

Recurrences are common, and these are usually in the pelvis, peritoneum, lymph nodes, and rarely into the other visceral organs such as the liver or lungs. Though these tumors are highly chemosensitive, management of recurrent disease according to the limited case series is by secondary cytoreductive surgery, salvage chemotherapy, or, sometimes, a combination of both [5].

Because of the rarity of these IOTs, management of the recurrent disease has been quite challenging. Thus far, debate exists about the management of recurrences and long-term outcomes after primary treatment. Additionally, many studies have focused on the paediatric and adolescent population, and very few studies have been conducted on women of all ages. This study aimed to review the clinical characteristics and to evaluate the oncological outcomes of immature ovarian teratomas following primary and secondary cytoreductive surgeries in a South Asian tertiary care center.

This article was previously presented as a meeting abstract at the 2nd Shalamar IRC RCOG summit on 14-16 Jan 2025, Lahore, Pakistan.

## Materials and methods

After obtaining approval from the Institutional Review Board of Shaukat Khanum Memorial Trust, a retrospective cohort study was performed in Shaukat Khanum Memorial Cancer Hospital and Research Centre, Lahore, Pakistan. Data were collected from the hospital cancer registry, and patient identifiers were anonymized. Women who were diagnosed as having IOTs and were treated at our hospital between July 2008 and June 2023 (15 years) were included in the study. Those women were excluded whose primary treatment was not in our hospital, and those who did not have long-term follow-up data in the hospital record.

Clinical characteristics included age, Federation of Gynecology and Obstetrics (FIGO) stage, and grade of the tumor. The route of surgery, whether laparoscopy or laparotomy, was decided by the preference of the surgeon and also by the size of the ovarian mass. The type of primary surgery included cystectomy, unilateral salpingo-oophorectomy, bilateral salpingo-oophorectomy, and total hysterectomy with complete staging in every case. After primary surgery, whether women need adjuvant chemotherapy or not is determined. Having a relapse is a very important question to be addressed, so how many women have had a relapse, and what treatment for relapse was given. In terms of follow-up data, whether the women were in relapse, remission, lost to follow-up after a certain period of time or deceased, was recorded. Recurrence-free survival (confirmed by imaging studies) and overall survival were also recorded.

Data were analyzed using Statistical Product and Service Solutions (SPSS, version 23; IBM SPSS Statistics for Windows, Armonk, NY). Patient demographics and disease characteristics were summarized using descriptive statistics. Oncological outcomes are measured by recurrence-free and overall survival. Recurrence-free survival and overall survival were calculated by the Kaplan-Meier method. Progression-free survival (PFS) is defined as the period from surgery to the recurrence of the tumor or the last patient contact. The overall survival (OS) is defined as the period from surgery to death or the last patient contact.

## Results

All 37 patients included in the study had a histopathological diagnosis of IOTs in our hospital records. Radiological evaluation was performed to predict the stage of the disease. All patients, whether undergoing primary/secondary cytoreductive surgery or chemotherapy, were discussed in the multidisciplinary team meeting before further planning of management.

The majority of the study population belonged to the age group of 21-30 years (N-16, 43.2%). There was one patient above 50 years of age, depicting that though rare but these tumors can still be present in older age groups. Stage 1A is the commonest presentation, but surprisingly, the majority of the cases were grade III (N-12, 32.4%). Laparotomy was done in 34 (91.1%) patients, which may be due to the large size of the tumor. Our data showed that fertility-preserving surgery was done in most of the women (N-27, 73%) because of the early presentation of these tumors. Due to the high grade of the tumors in the majority of the women, 73% (N-27)of the women received adjuvant chemotherapy, as shown in Tables 1-2.

**Table 1:** Clinical characteristic of the study population

Variables		N (%)
Age (years)	0-10	6 (16.2)
	11-20	8 (21.6)
	21-30	16 (43.2)
	31-40	6 (16.2)
	41-50	0
	>50	1 (2.7)
Stage	1A	18 (48.6)
	1C	3 (8.1)
	III	2 (5.4)
	IV	1 (2.7)
	Unknown	13 (35.1)
Grade	I	9 (21.6)
	II	8 (24.3)
	III	12 (32.4)
	Unknown	8 (21.6)

**Table 2:** Type of treatment given

Variables		N (%)
Route of surgery	Laparoscopy	2 (5.4)
	Laparotomy	34 (91.9)
	Surgery not performed	1 (2.7)
Type of surgery	Cystectomy	3 (8.1)
	Unilateral salpingo-oophorectomy with staging	27 (73.0)
	Bilateral salpingo-oophorectomy with staging	2 (5.4)
	Total hysterectomy, bilateral salpingo-oophorectomy with staging	4 (10.8)
	Surgery not performed	1 (2.7)
Adjuvant chemotherapy	Yes	27 (73)
	No	10 (27)
	Treatment for relapsed cases (13)	Surgery	8 (61.5)
Chemotherapy		5 (38.4)

Relapse occurred in 13 (35.1%) cases, and the sites of relapse were the pelvis in four cases (10.8%), omentoperitoneum in three cases (8.1%), liver in five cases (13.5%), and bones in one case (2.7%). Surgery was done in eight (61.5%) women for relapsed disease, whereas chemotherapy was given in the rest of the cases.

**Figure 1:**
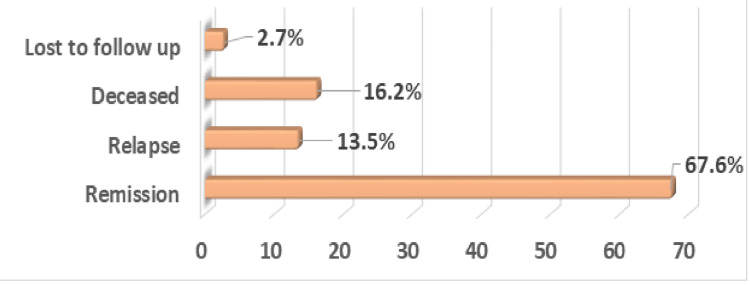
Long-term follow-up of the study population

**Figure 2:**
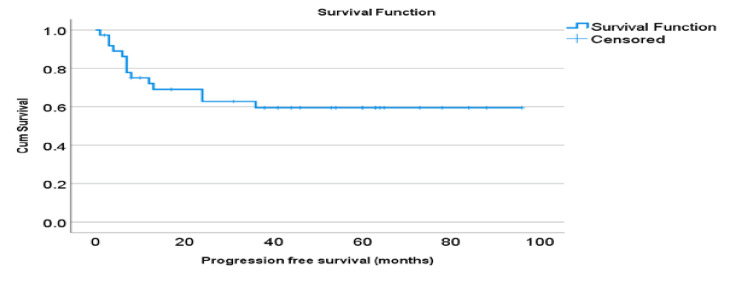
Progression-free survival (PFS)

**Figure 3:**
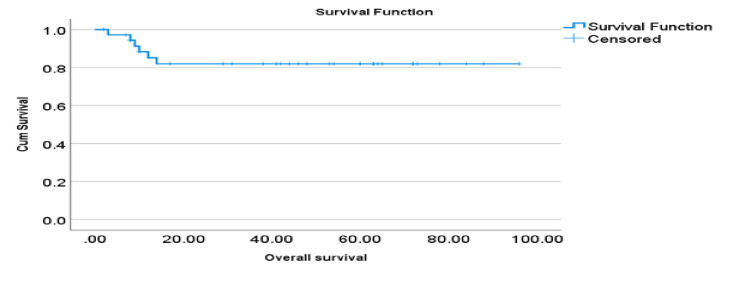
Overall survival in months

As described in Figure 1, the majority of the women were in remission on follow-up data (N-25, 67.6%). However, 16.2% (N-6) of the women were deceased, 13.5% (N-5) were in relapse, and only 2.7% (N-1) did not have long-term follow-up data. Progression-free survival was recorded to be 60%, and overall survival was 81% over the period of 60 months, as shown in Figures 2-3.

## Discussion

The small cohort of patients (n = 37) over a period of 15 years in our dedicated cancer center represents the rarity of IOTs. Our study confirms that IOTs affect the younger age group commonly, but they are usually higher grade, requiring adjuvant chemotherapy after surgery. Overall, progression-free and overall survival are good. In this study, we have focused on prognostic factors, which are tumor grade, stage, completeness of surgical staging, and adjuvant chemotherapy.

Our study has shown that IOTs are common in the younger age group, which aligns with the published data [6]. It affected mostly the age group of 20-30 years in our study. There was only one patient who was above 50 years, which highlights that there is a possibility of these tumors in the older age group, so that differential diagnosis should be kept in mind. The finding that the majority of the women presented early in the disease mirrors the large cohort of other studies where >80% of the women presented early [6]. A higher grade of the disease in 32% of our study cohort is consistent with the global data [7]. Pashankar et al. also highlighted that grade III disease affected >56% of their study population [7].

Laparotomy was the main surgical approach (91.9%), which may be because of the large size of the tumor masses. Despite such large masses, the fertility sparing technique (unilateral salpingo-oophorectomy with staging) was employed in 73% of the cases, which is according to the European Society for Medical Oncology (ESMO) guidelines [3,8]. Wang et al. demonstrated that there was no difference in PFS and OS in terms of fertility sparing surgery or radical surgery, so there is no need to do a radical surgery in these cases [8].

Adjuvant chemotherapy was required in 73% of our cases, which is due to the higher grade of the disease. Several studies have demonstrated that adjuvant chemotherapy in grade >2 disease has significantly reduced the mortality rate (RR: 0.17 for recurrence, RR: 0.31 for mortality), suggesting that, even if it is early disease, adjuvant chemotherapy should be considered in high-grade disease [9]. A recurrence of 35% in our study indicates a higher chance of recurrence, which may be because of the high-grade disease. A study by Terenziani et al. has reported higher recurrence rates in grade 2 and 3 disease, but they were very well managed by secondary cytoreductive surgeries in the majority of the cases. In our study, secondary cytoreductive surgery was done in 61.5% of the cases, which is in line with the study from western China, where secondary cytoreductive surgery for relapsed disease significantly increased PFS [9,10].

Our long-term follow-up data showed RFS of 60% and OS of 81% which is lower than previous large retrospective studies. The western China cohort demonstrated a PFS of 74.3% and an OS of 96.5%, whereas Li et al. highlighted a PFS of 86%. This may be because of the higher grade of the disease in our study cohort [6,10]. Though our study followed the patients for their fertility outcomes, published literature has reported good fertility outcomes exceeding 80%. Wang et al. highlighted successful pregnancy outcomes in 5/7, even in advanced-stage disease cases treated by surgery [8].

Strengths of our study include a longer study period, and it actually reflects real-world data from the South Asian region. A single-center study could mean that patients were managed by uniform protocols, including multidisciplinary input in all cases as per hospital protocol. Limitations include its retrospective study design; a smaller sample size because of the rarity of these tumors; missing data on stage/grade of tumor; and a lack of follow-up data for fertility outcomes.

## Conclusions

Our study reinforces the individualized management of IOTs with respect to the stage and grade of the disease. Fertility-sparing surgery in early-stage disease with adjuvant chemotherapy has promising long-term survival outcomes, though a high grade of the disease is challenging. A large number of patients with recurrent disease can be treated successfully with secondary cytoreductive surgery or salvage chemotherapy. Future studies can include fertility outcomes in early and advanced stage disease to better guide this cohort of women. Though a rare tumor, IOTs should always be considered in the differential diagnosis of adnexal masses in young women.
